# In situ characterization of nanoscale contaminations adsorbed in air using atomic force microscopy

**DOI:** 10.3762/bjnano.9.271

**Published:** 2018-11-23

**Authors:** Jesús S Lacasa, Lisa Almonte, Jaime Colchero

**Affiliations:** 1 Centro de Investigación en Óptica y Nanofísica (CIOyN), Departamento Física, Facultad de Química, Campus Espinardo, Universidad de Murcia, 30100 Murcia, Spain; 2 Electrical Engineering and Biological Science, University of Notre Dame, Notre Dame, Indiana 46556, USA

**Keywords:** atomic force microscopy, cantilever, contact potential, electrostatic forces, force spectroscopy, Hamaker constant, Kelvin probe microscopy, surface contamination, tip cleaning, tip–sample interaction, van der Waals interaction

## Abstract

Under ambient conditions, surfaces are rapidly modified and contaminated by absorbance of molecules and a variety of nanoparticles that drastically change their chemical and physical properties. The atomic force microscope tip–sample system can be considered a model system for investigating a variety of nanoscale phenomena. In the present work we use atomic force microscopy to directly image nanoscale contamination on surfaces, and to characterize this contamination by using multidimensional spectroscopy techniques. By acquisition of spectroscopy data as a function of tip–sample voltage and tip–sample distance, we are able to determine the contact potential, the Hamaker constant and the effective thickness of the dielectric layer within the tip–sample system. All these properties depend strongly on the contamination within the tip–sample system. We propose to access the state of contamination of real surfaces under ambient conditions using advanced atomic force microscopy techniques.

## Introduction

Surface science is fundamental to understand many processes in industrial applications, environmental science, biology, medicine and phenomena such as self-assembly [1], friction [2–3] and wetting [4]. In any study involving surfaces a well-defined surface condition is essential. Preparing such surfaces has thus been a field of intense research over many decades. One approach is to work under ultrahigh-vacuum (UHV) conditions, which has opened fascinating experimental possibilities [5]. More importantly, well-defined surfaces allow for a detailed comparison between theory and experiments triggering an immense advance of surface chemistry.

While UHV conditions are experimentally and theoretically appealing, most processes occur under ambient conditions, where it is much more difficult to prepare well-defined surfaces. In ambient air, surfaces are covered by a variety of molecules and nanoparticles that drastically modify its properties as compared to ideal and clean surfaces. Quite a few fields ranging from fundamental studies of wetting phenomena [6] to semiconductor industry [7] are very aware of the importance of clean and well-prepared surfaces. Accordingly, a wealth of experimental techniques have been developed to control and characterize their contamination state [8]. In the present work we propose atomic force microscopy (AFM) [9–10] as a valuable tool to visualize nanoscale surface contamination and to quantify its physical properties.

The AFM tip–sample system can be considered on the one hand a model system for investigating different nanoscale phenomena, and a nanoscale “laboratory on the tip” on the other [11]. AFM operation is based on the interaction between a sharp tip and the sample to be analyzed. To obtain images, this interaction is maintained constant by changing the normal position (*z*-direction) as the tip is scanned over the surface (lateral, *x*,*y*-directions). In spectroscopy applications, the lateral position is kept constant as the normal position of the sample is varied in order to access material properties (“chemical information”, thus the name spectroscopy) [12]. AFM allows not only the measurement of surface topography, but also the determination of other physical characteristics; in particular electrostatic [13–15] and magnetic properties [16–17].

For reliable data acquisition a well-defined and stable tip is essential. Therefore, numerous efforts have been devoted to fabricate tips that are well characterized and at the same time as sharp as possible [18]. However, even the best tip as fabricated originally may be of limited use if it does not maintain its specified properties during AFM operation [19–20]. Tip degradation, either tip wear or tip contamination from the sample, is mainly induced by AFM operation [18,20–21]. Other kinds of contamination that may affect the tip are organic contamination from ambient air, metallic pollutants due to the manufacturing processes, as well as contamination induced by the Gel-Pak containers employed for chip transport and storage [22–23]. A variety of cleaning procedures have been proposed to recover the ideal tip as manufactured initially. These procedures range from less aggressive methods, such as washing with sodium dodecyl sulfate (SDS), alcohols (e.g., ethanol) or acetone [24], ultraviolet (UV) [25] and ozone treatment [26], imaging of gratings [27–28], heating to evaporate contaminants [29–30], argon bombardment and even ultrasound, to much more aggressive methods, such as cleaning in piranha solution [31] and the RCA process [32], both of which are very successful in the removal of organic and metal contamination.

Several methods for tip characterization have been proposed, such as X-ray analysis, Raman spectroscopy, contact angle measurements, and scanning and transmission electron microscopy [33–36]. Since the tip may change quite frequently during AFM operation, ex situ methods may be quite inadequate and unproductive for many AFM experiments. Some methods have been proposed to infer tip properties, in particular the radius, using AFM techniques, which in our opinion is the optimal approach [37–40].

As discussed above, AFM is fundamentally based on tip–sample interaction. In this context, we may interpret that the tip is one half of the system, the sample being the other half. Unfortunately, the tip is the half of the system that is not directly seen, making tip characterization using AFM techniques a non-trivial task. To formalize this idea, we recall that within the Derjaguin approximation, the tip–sample force *F*_n_ is [41]:

[1]
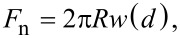


where *w*(*d*) is the interaction energy per unit area of two infinite surfaces separated by a distance *d*, and *R* is the tip radius (more precisely, *R* is the effective radius of the tip–sample system; 1/*R* = 1/*R*_tip_ + 1/*R*_surf_ with *R*_tip_ being the tip radius and 1/*R*_surf_ being the local surface curvature). Equation 1 shows that the tip–sample force depends on the tip geometry (described by the tip radius) on the one hand, and on the materials properties on the other (described by the surface energy *w*(*d*)). In the context of the present work we note that Equation 1 may be understood in the following way: it separates the tip–sample interaction into a term describing the geometry (radius *R*) and a term *w*(*d*) describing the chemistry of the system.

In the present work, we propose to characterize the tip system following two basic ideas. First, we will focus on topographic imaging, and we will show that the contamination state of the tip can be (indirectly) imaged by assuming that, to a first approximation, the apex of the tip should have a similar degree of contamination as the rest of the cantilever and as the chip onto which the tip and cantilever are attached. Second, we will assume that by precisely measuring the tip–sample interaction we can infer properties related to the surface energy as well as the contact potential, which allows one to access the chemistry of the tip–sample system.

## Results and Discussion

### Topographic imaging of the tip and the flat part of a cantilever

**Figure 1:**
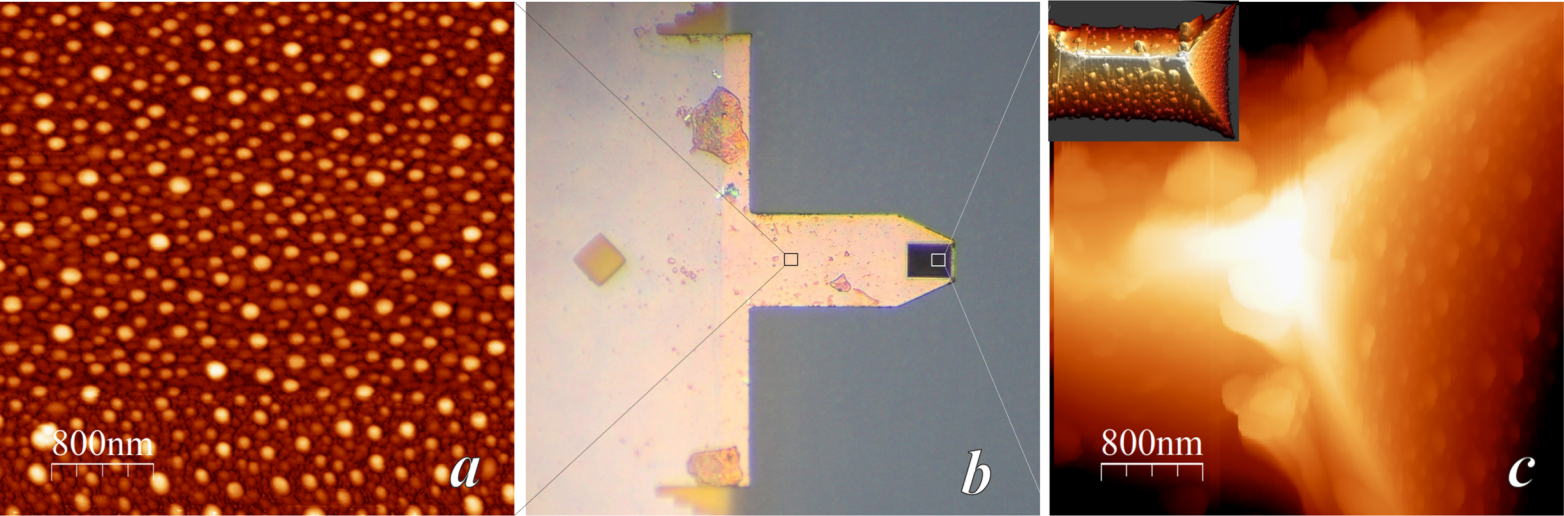
AFM images (a, c) and optical image (b) of the tip side of an Olympus OMCL-HA-100 AFM cantilever. Image sizes: 4 × 4 μm^2^ and 80 nm height scale for the AFM image in panel (a) showing the flat region of the cantilever; about 300 × 300 μm^2^ for the optical image in panel (b); and 4 × 4 μm^2^ and 2 µm height scale for the AFM image in panel (c) showing the tip region of the cantilever. The inset in the right AFM image shows a 3D representation of (almost) the whole cantilever tip. The dotted squares in the optical image mark the two regions where the AFM images have been acquired.

Figure 1 shows images where the lower side of the cantilever, that is, the side with the sensing tip, has been used as the sample. As discussed below, tip imaging can be experimentally very challenging, due to very large tip–sample interaction. In spite of these problems, we have been able to image an AFM cantilever and its tip. Figure 1 shows the flat cantilever part (Figure 1a), an optical image of the whole cantilever (Figure 1b) and the tip (Figure 1c). In the optical image the scan areas corresponding to the two AFM images are marked with two rectangles. The sample cantilever was taken out of the box and imaged without further processing (in particular no cleaning). As can be clearly observed in Figure 1a, the cantilever surface is covered by round islands of typically 50 nm diameter and 10–20 nm height (contact angle of these drop-like particles assuming a spherical surface: about 45°). From this topographic image we deduce that the surface of the cantilever is severely contaminated. Moreover, as observed in Figure 1c, essentially the same kind of contamination is observed on the flat part of the cantilever and on the sides of the tip cone. Even though we have not been able to image the tip apex at high resolution we believe that our observation supports the hypothesis that if the composition of the whole cantilever is uniform then the contamination observed on the flat part of the cantilever is analogous to the contamination present on its tip. We assume that the contamination layer behaves like a carpet covering all parts of the cantilever, and in particular also the tip apex used as probe in AFM applications. We conclude that if the material of the whole cantilever is uniform, then the contamination state of the tip can be inferred by characterizing the flat part of the cantilever, which is the experimentally much simpler system.

As noted above, imaging of the tip is quite difficult. In this context we note that tip–sample interaction between the lateral sides of the tip may be huge if two plane sides of the probe-tip and the sample-tip interact together. In a sense, such a tip-probe versus tip-sample system has an infinite effective radius *R* resulting in a huge adhesion force (*F*_adh_* =* 4π*R·*γ·cos(φ), with γ being the surface energy of water and φ being the contact angle of the (nano-)meniscus within the tip–sample system. Usually, the system “wets”, then cos(φ) ≈ 1). In our experiments we have observed with an optical microscope how the probe-tip jumps into contact with the sample-tip. Then, the whole probe system, i.e., the mesoscopic cantilever and the macroscopic chip to which it is attached (typical size 2 × 4 mm^2^), is moved by the scanning motion of the piezoelectric element.

### Topography and electrostatic images of cleaned and uncleaned cantilevers: accessing materials properties

**Figure 2:**
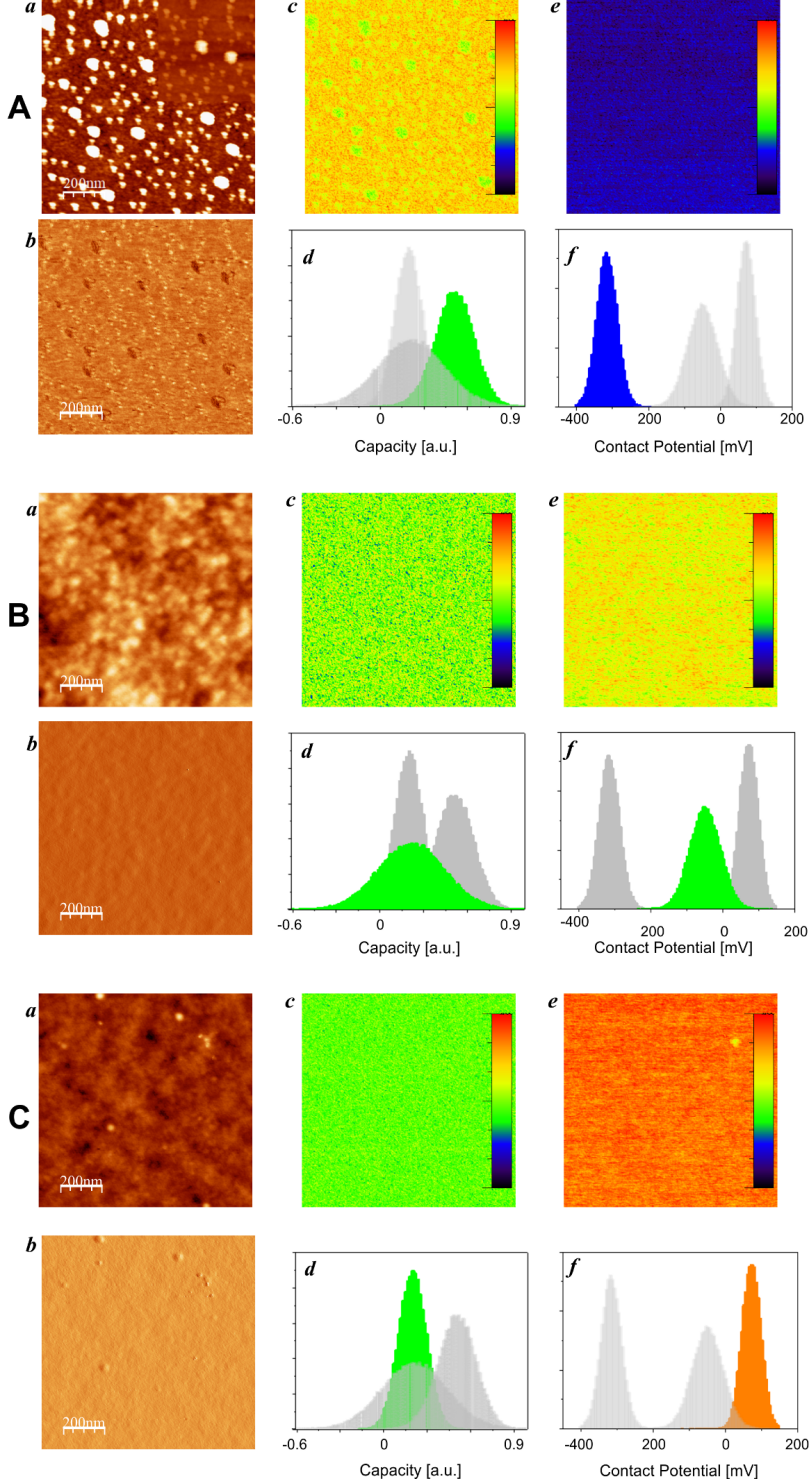
Topography (*a*), error signal (*b*), capacity data (*c*) and contact potential (*e*) acquired on the flat part of three platinum-coated AFM cantilevers: “uncleaned-old“ (panel A), “uncleaned-new” (panel 2B) and “cleaned” (panel C). Image size is 1 × 1 µm^2^. Height scale for all topography images is 5 nm (with the exception of the right top inset in Figure 2A*a*, where it is 20 nm), the voltage range is −600 to +100 mV for the contact potential images, and arbitrary units (output of lock-in amplifier) are given for the capacity images. The histograms (*d*) and (*f*) show the variation of data in the corresponding images above, namely capacity (*c*) and contact potential (*e*). We note that data is shown in “logic order”, rather than ordered by time, in order to avoid contamination from the sample to the tip. The chronological order in which these images were acquired is: first the sample “uncleaned-new”, then (RCA) “cleaned”, and finally “uncleaned-old”.

Topographic images as shown in Figure 1 are quite valuable if the sample is well controlled. However, in many cases the morphology gives only a limited amount of information with respect to the chemical composition of the sample. Fortunately, AFM provides additional “secondary channels” from which compositional information can be extracted. As discussed in more detail elsewhere [42], data can be acquired in the (true) non-contact regime (nc-DAFM), where only van der Waals and electrostatic interaction is present. In this work we will assume that the tip–sample system can be described by a metallic tip interacting electrostatically with a metallic sample and that on each metallic surface a thin dielectric film may be adsorbed. In addition, we will assume that the (second derivative of) tip–sample capacitance (see Equation 2) can be approximated by the expression *C*″(*d*) = πε_0_*R*/(*d + h*/ε)^2^, where *R* is the effective tip radius, *d* is the tip–sample distance, *h* is the total thickness of the dielectric films on tip and sample, ε_0_ is the dielectric permittivity of vacuum and ε is the relative dielectric constant [43–46]. For a purely metallic system in air or vacuum were no dielectric layer is present (*h* = 0), the expression simplifies to *C*″(*d*) = πε_0_*R*/*d*^2^. Then, the total frequency-shift induced by the tip–sample interaction is:

[3]
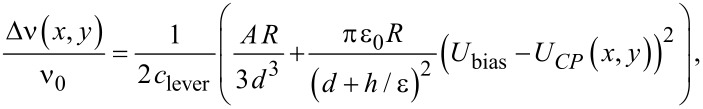


where the first term containing the Hamaker constant *A* describes the van der Waals interaction and the second term describes the electrostatic interaction. We note that the chemical composition of the sample will determine three different parameters in this relation: the Hamaker constant *A*, the contact potential *U*_CP_ and the pole *d*_pole_ = −*h*/ε of the capacity term. All three terms will be used in the present work to infer the composition of the tip–sample system. Figure 2 shows images of three different experiments where the flat part of the cantilever chip has been analyzed in (true) nc-DAFM using Kelvin probe microscopy (KPM) to measure the contact potential. The three analyzed samples correspond to the surface of platinum-films evaporated onto silicon cantilevers, but with three different state of contamination: One was taken from a box that had been opened for the first time quite a long time ago (more than a year) and will be termed “uncleaned-old” (Figure 2A). Another one was taken from a freshly opened box and will be termed “uncleaned-new” (Figure 2B). The last one, which will termed “cleaned” (Figure 2C), has been cleaned just before imaging using the RCA procedure discussed in the Experimental section. The same probe-tip was used for all experiments. This tip was taken from a freshly opened cantilever box and used without further processing. In the nomenclature of the present work the probe-tip was thus of type “uncleaned-new”, which is “almost clean”, as discussed below in more detail.

Figure 2 shows the topography (*a*), the error signal of the feedback (frequency shift, (*b*)), the electrostatic capacity signal (EAFM_2ν_, see Experimental section, (*c*)) as well as the contact potential (*e*), which are all measured simultaneously. We will first discuss the results obtained for the sample “uncleaned-old”. For the cantilever imaged in Figure 1, the topographic image (Figure 2A*a*) shows round islands of approximately 50 nm diameter and 10–20 nm height, which we again associate to surface contamination. We note that although the surface composition of the cantilever imaged in Figure 1 (material: silicon nitride) and that analyzed in Figure 2A (material: Pt-coated silicon) is very different, the topography of the surfaces looks quite similar. This seems reasonable taking into account that both cantilevers came from a box that had been opened long time before the experiments. Therefore, these cantilevers had enough time to be exposed to ambient air (each time a cantilever is extracted from the box) and become severely contaminated.

From the topography image (Figure 2A*a*) one would conclude that the platinum surface is covered by some contamination (round islands); that is, one would expect two kinds of materials on the surface: the platinum substrate and some other most probably organic material on top of the platinum. Surprisingly, the contact potential image (Figure 2A*e*) is completely homogeneous, leading to the conclusion that either the islands are completely transparent to the measurement of contact potential or that the platinum surface is covered everywhere by the same material, that is, the contact-potential image detects the same material on the islands as well as on the flatter part between the islands. Since the contact potential image is very sensitive to differences in work function and since it seems unlikely that platinum has the same work function as the (unknown) material of the island, we think that the most logical explanation for the homogeneous contact potential image is that the whole surface is covered by the same material, which corresponds to an adsorbed contamination layer. This explanation is also supported by results discussed below.

In contrast to the contact-potential image, the capacity image (EAFM_2ν_, Figure 2A*c*) shows a clear contrast between the lower region and the islands. According to Equation 3, the capacity term is *C*″(*d*) = πε_0_*R*/(*d + h*/ε)^2^*.* We therefore interpret that the lower capacity on the islands is due to a larger thickness *h*/ε of the (dielectric) contamination film. Note that if the islands were composed of a conducting material, then *h* = 0 and we would expect no contrast of the capacity *C*″(*d*).

Figure 2B shows the results obtained for the cantilever of type “uncleaned-new”, i.e., a cantilever that had been taken from a freshly opened cantilever box. In this case, all images, topography, contact potential and capacity, appear quite homogeneous. Unfortunately, this homogeneity does not indicate whether the surface is homogeneously clean, or homogeneously contaminated.

Finally, Figure 2C shows images corresponding to a cantilever cleaned using the RCA method. The topography image (Figure 2C*a*) is, on a larger scale (200 nm) quite flat showing a small irregular corrugation (2 nm), which is typical of the silicon substrate on which the thin platinum film (nominally 20 nm thickness) is evaporated. On a smaller scale (image not shown) this sample shows the rounded structure of the polycrystalline platinum grains (ca. 20 nm lateral size, about 1 nm mean roughness, see for example [47]). A few higher structures (3–4 nm height) can be recognized in the topographic images. Interestingly only one of these higher structures gives a contrast in the contact-potential image (Figure 2C*e*, −90 mV as compared to +70 mV for the substrate), otherwise the contact-potential image is completely homogeneous. We interpret this data as follows: Almost the whole sample has been effectively cleaned, excluding the higher structure with a different contact potential value, which corresponds to some material other than platinum.

### Multidimensional AFM spectroscopy of cleaned an uncleaned cantilevers

**Figure 3:**
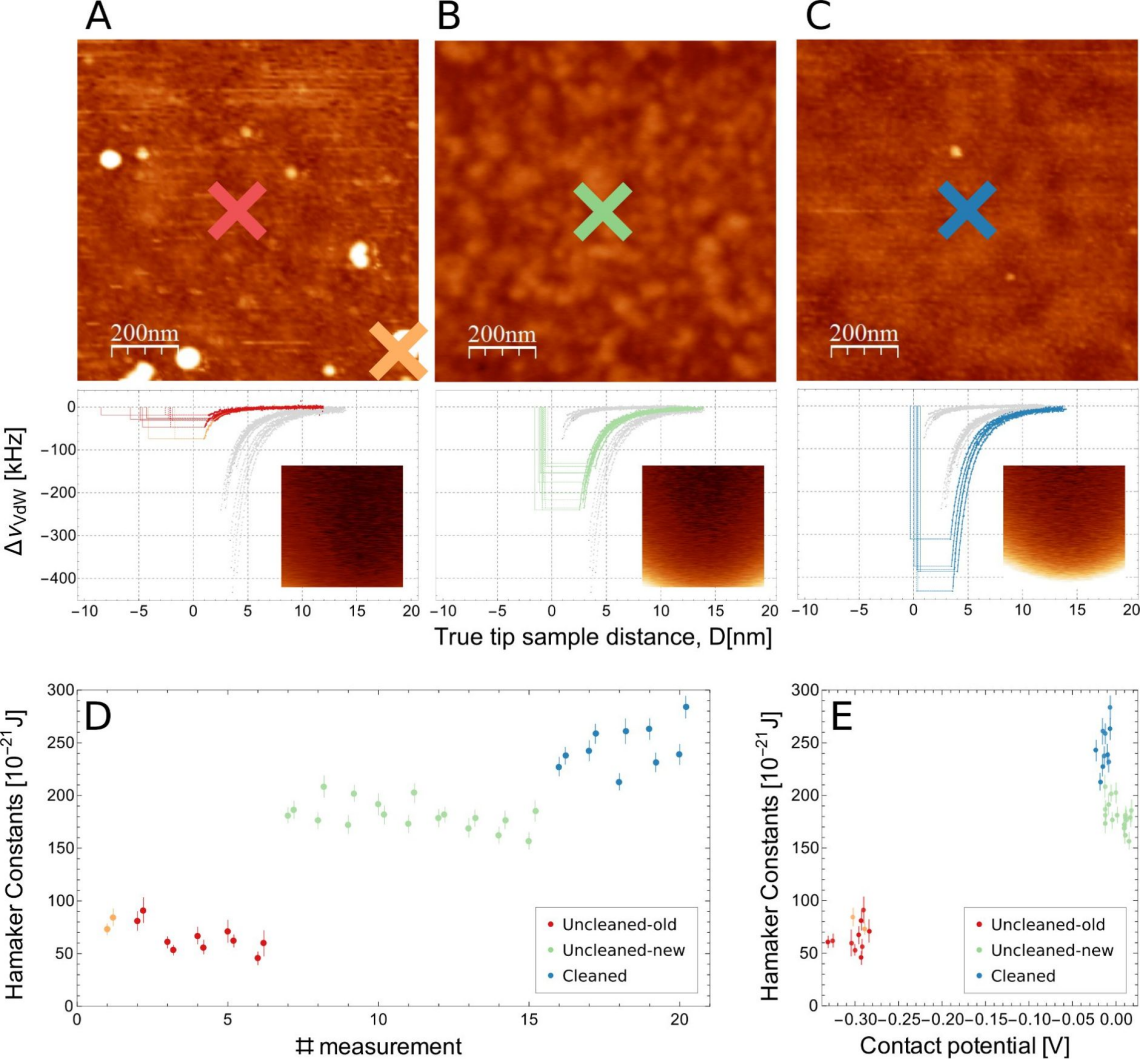
Topography images and spectroscopy data at specific locations on three different Pt-coated cantilever chips used as samples. Each chip has a different degree of contamination: “uncleaned–old” (column A), “uncleaned–new” (column B) and “cleaned” (column C). Top row: topography images. Middle row: van der Waals spectroscopy curves Δν_vdW_(*d*). The different curves are shown in the color corresponding to the cross in the topographic images above marking the position of the spectroscopy experiment. Note that in each graph all curves are shown; those at the position marked in the topographic image above are shown in colour, while all the other curves are shown in grey for comparison. The tip–sample distance corresponds to the true tip–sample distance, i.e., *d* = 0 corresponds to the positon of the surface as seen by the van der Waals interaction. The vertical lines to the left (negative distance) of the experimental Δν_vdW_(*d*) curves in these graphs show the electrostatic pole (see main text). The inset in these graphs show one of the interaction images (raw data, total color scale 300 Hz, the horizontal scale (fast scan, bias voltage) corresponds to ±0.8 V and the vertical scale (slow scan, tip–sample distance) corresponds to 10 nm. The Δν_vdW_(*d*) curves are calculated from these interaction images. Bottom row: Hamaker constant (D) and Hamaker constant versus contact potential diagram (E) for all spectroscopy experiments acquired at the positions shown in the different topographic images. The colors of the data points correspond with the positions of the different data sets (and samples) marked in the topographic images.

To better characterize the tip–sample system, we have acquired multidimensional spectroscopy data, so-called interaction images, at specific locations of the sample. Figure 3 shows the results of experiments in which (topography) AFM imaging is combined with the acquisition of interaction images at well-defined spots. AFM data, topography images and interaction images, were acquired on all types of samples. In interaction images the horizontal axis (fast scan direction) corresponds to a voltage sweep, while the vertical axis (slow scan direction) corresponds to the tip–sample distance [48]. The color scale shows the variation of resonance frequency due to tip–sample interaction as a function of bias voltage and tip–sample distance (see insets in the Δν_vdW_(*d*) graphs of Figure 3). These interaction images are then processed to separate van der Waals and electrostatic interaction. For each interaction image three curves are obtained: a (true) van der Waals interaction Δν_vdW_(*d*) curve (Figure 3, middle row), a tip–sample capacitance *C*″(*d*) curve (not shown) and a contact potential *U*_CP_(d) curve (not shown). As discussed in the Experimental section, by fitting these curves to the relation that describes their variation as a function of the distance (Equation 3), three material-specific parameters can be obtained: the Hamaker constant *A*, the thickness of the dielectric layer *h*/ε and the contact potential *U*_CP_.

On each spot, several interaction images were obtained, the corresponding results are shown in Figure 3D and summarized in Table 1. In total 20, spectroscopy experiments (*n* = 1–20) have been performed on the three different samples. For each single experiment two data sets are acquired, processed and shown: one corresponding to an approach cycle and the other one corresponding to a retraction cycle of a spectroscopy experiment. Data sets *n* = 1–6 are obtained on an “uncleaned-old” cantilever, data sets 7–15 on an “uncleaned-new” cantilever and data sets *n* = 16–20 on a (RCA) “cleaned” cantilever. From each spectroscopy experiment the Hamaker constant *A* (Figure 3D) the contact potential *U*_CP_ and the thickness of the dielectric layer *h*/ε_0_ were determined; the corresponding mean values of these parameters are listed in Table 1.

**Table 1:** Hamaker constant, contact potential and thickness of the dielectric layer obtained by processing interaction images on three samples with different contamination states.

sample	Hamaker constant *A* [10^−21^ J]	contact potential *U*_CP_ [V]	dielectric layer *h*/ε_0_ [nm]

“uncleaned-old”	66 ± 8	−0.30 ± 0.02	5 ± 8
“uncleaned-new”	180 ± 12	0.00 ± 0.01	1 ± 1
“cleaned”	245 ± 20	−0.02 ± 0.01	0 ± 0.5

All data is acquired in the true non-contact regime, that is, the tip–sample system is neither in mechanical contact, nor are liquid necks formed [42]. In fact, the oscillation amplitude during all experiments was the free oscillation amplitude, which implies neither snap to contact nor sudden decrease of oscillation amplitude due to formation of liquid necks (see discussion and Figure 4 in [48]). Correspondingly the data is very reproducible and we observe a clear difference between data acquired on the samples with different degrees of contamination. Clearly, the difference of the values obtained for the Hamaker constant, the contact potential and the thickness of the dielectric layer obtained for each individual spectroscopy experiment is smaller than the difference of these values obtained on the different cantilevers. This is most easily visualized in the plot of the Hamaker constant as a function of the contact potential (Figure 3E) where the measurements can clearly and uniquely be attributed to the three different groups. We are therefore able to measure a variation of the Hamaker constant, as well as of the contact potential, due to the chemical differences induced by the contamination in the tip–sample system.

From the data shown in Figure 3D and the mean values for the Hamaker constant listed in Table 1 we conclude that the effective Hamaker constant of the system decreases when the tip–sample system is more contaminated. This, in our opinion, is easily understood in terms of the higher Hamaker constant of platinum compared to that of organic contaminants, which most likely constitute most of the contamination adsorbed on the tip and on the sample. In this context we note that our methodology gives the correct value of the Hamaker constant of the platinum–platinum system *A*_Pt_ = (245 ± 22) × 10^−21^ J when the system has been (RCA) cleaned and immediately imaged. A lower, but still high (metallic) Hamaker constant *A*_eff_ = (180 ± 12) × 10^−21^ J is obtained for the cantilever of type “uncleaned-new”, and a very low Hamaker constant *A*_eff_ = (66 ± 8) × 10^−21^ J is obtained for a highly contaminated (“uncleaned-old”) tip–sample system. Interestingly, in this case essentially the same Hamaker constant is obtained on the flat part of the sample and on one of the drop-like islands, which quite clearly correspond to contamination. We therefore conclude that on these latter samples the contaminating film should be quite thick so that the van der Waals interaction with the platinum below the contamination is negligible.

Interestingly, we are able to detect not only the variation of Hamaker constant and contact potential due to the contamination, but also the variation of the pole of the interaction (see Equation 3). The algorithm to process the interaction images determines not only the tip radius from the term *C*″(*d*) = πε_0_*R*/(*d + h*/ε)^2^. It also determines the poles of the electrostatic and the van der Waals interaction. The data in the van der Waals versus distance curves Δν_vdW_(*d*) shown in Figure 3 have been normalized so that the position of the surface obtained from the van der Waals curve is at *d* = 0, and the electrostatic pole (that is, the position where the electrostatic interaction "sees" free electrons) is at the vertical lines shown for each curve that has been processed. A vertical line that is at a large negative (= left) distance therefore corresponds to a large *h*/ε term due to a dielectric film covering the metallic Pt surface. We thus conclude, again, that the sample “uncleaned–old” must be homogeneously covered by a non-metallic contamination layer of thickness *h*/ε, in complete agreement with the conclusion drawn from the electrostatic images shown in Figure 2. For the sample of type “uncleaned–new” the thickness *h*/ε is about 1 nm while for the sample “cleaned” the electrostatic pole coincides with the van der Waals pole at *d* = 0.

## Conclusion

The present work describes our effort to shed light on the issue of nanoscale contamination on surfaces, and in particular within an AFM tip–sample system that is used here on the one hand as a model system, and on the other as a characterization instrument. It is generally accepted that contamination must be present when working under ambient conditions. Otherwise the extreme effort to work under UHV conditions would not make any sense. However, in general the precise way in which this contamination affects many surface processes is neither understood in detail nor well controlled. In fact, in our opinion, this issue is often just ignored, in particular in the AFM community. Many more AFM related measurements are performed under ambient conditions as compared to UHV, and AFM under ambient conditions has become a fundamental and widely used tool for nanoscale research in materials science, physics, biology and medicine, chemistry as well as engineering and environmental sciences. We are therefore convinced that this issue of quantification of surface contamination under ambient conditions is not merely a “technical issue” related only to AFM operation, but rather a topic of much broader relevance.

From our work we conclude that not only contamination of the tip, but also that on the surface is quite difficult to visualize and detect, but is nevertheless quite important, and significantly determines AFM results and interpretation. In the present work we have shown how the presence of contamination in the tip–sample system can be detected and quantified using only AFM techniques. The methodology presented allows for simple in situ characterization and checking of the tip–sample system, without needing to remove the tip and/or the sample from the AFM setup.

To infer the contamination of the tip we have adopted the assumption that, if the whole cantilever is composed of the same material then the contamination should cover the tip apex, the tip and the flat part of the cantilever in a similar way. This assumption should be quite correct if the contamination layer is thinner than the radius of curvature of the tip, typically 20 nm, which should be the case in most AFM experiments. Taking two equivalent cantilevers and using one as probe and the other one as sample allows one to observe and measure the properties of the tip, since in a sense, the tip is looking at a (statistically) equivalent sample system. Within the Derjaguin approximation (see Equation 1) the chemistry of the tip–sample system is described by the surface energy *w*(*d*) and we interpret that for the Pt–Pt tip–sample system used in the present work this surface-energy term is due to twice the same system: in our case Pt with a (dielectric) contamination layer.

To validate our assumption, we have first imaged the tip and the flat cantilever regions of a cantilever taken from a typical enclosing box. As discussed previously in the literature, we find a high degree of contamination on the cantilevers and, more importantly in the present context, we observe that this contamination layer covers in a similar way the tip and the flat part of the cantilever (Figure 1). In the second experiment we have used imaging of topography as well as of electrostatic properties to characterize cantilevers used as a sample. We have imaged three tip–sample systems with different degrees of contamination, and find different electrostatic responses of the capacity signal and of the contact potential (Figure 2). Unfortunately, the data obtained from these images is not completely conclusive: A contact potential difference close to zero may indicate that the tip and the sample are either clean, or that both are contaminated in a similar way. Using multidimensional spectroscopy techniques (interaction imaging) and advanced data processing we are capable to locally determine the effective Hamaker constant, the contact potential and the thickness of the dielectric layer of the tip–sample system; and we find that in particular the Hamaker constant is very sensitive to the contamination state of the tip–sample system (Figure 3). Finally, we note that the methodology of acquisition and processing of interaction imaging is independent of the tip radius, an important parameter for tip–sample interaction (see the Derjaguin approximation, Equation 1) that is often quite difficult to obtain precisely. In fact, we believe the precision and robustness of our methodology is due to the fact that its results do not depend on the tip size.

We propose the method presented in this work as a simple, very precise and sensitive, and, very important, in situ characterization of the AFM tip–sample system. Moreover, we believe that our methodology is particularly suited for AFM since it is based on determining the effect of contamination on the tip–sample interaction, which is the fundamental physical property that determines AFM operation. That is, as compared to other non-AFM-based techniques for the determination of contamination, our method detects the contamination that particularly affects the tip–sample interaction, and that will therefore have an important effect on AFM data acquisition and data interpretation. In the future this method will be easily extended to improve nanoscale materials characterization in the following way: Once the tip is known to be clean, the properties of an unknown surface can be characterized as discussed in the present work precisely because the tip side is clean and well controlled and not anymore an unknown parameter of the system. Only then the measured properties of the tip–sample system can be uniquely attributed to the sample.

## Experimental

### AFM data acquisition and processing

#### AFM imaging

Data was acquired using dynamic atomic force microscopy (DAFM) on a Nanotec Electronica AFM system with a phase-locked loop board (PLL, bandwidth ca. 2 kHz), which maintained the cantilever at resonance. Images and spectroscopy were acquired using the frequency as signal for the feedback channel (frequency-modulation dynamic mode; FM-DAFM [49]) at small oscillation, which generally implies non-contact operation (so-called attractive regime), for more detail see previous works of our group [42,50–52]. The tip–sample distance is estimated to be between 5 and 10 nm, ensuring low-noise imaging with high spatial resolution, not only of topography but also of electrostatic interaction (electrostatic resolution is estimated to be ca. 20 nm). Platinum-coated silicon tips (ν_0_ ≈ 70 kHz) with a nominal force constant of 3 N/m were used. The nominal radius value for tip apex of these probes is specified as 15 nm by the manufacturer.

As described in [50–51] electrostatic measurements were performed by detecting the frequency shift (and thus the force gradient) induced by an alternating bias between tip and sample (also termed FM detection of electrostatic force). This frequency shift is:

[2] $$\frac{\Delta \nu_{\text{el}}\left(x,y\right)}{\nu_{0}}=\frac{C^{\prime \prime }\left(d\right)}{4\;c_{\text{lever}}}{\left(U_{\text{bias}}-U_{\text{CP}}\left(x,y\right)\right)}^{2},$$

where C″(*d*) is the second derivative of the capacitance, ν_0_ is the (free) resonance frequency and *c*_lever_ is the spring constant of the cantilever. For a bias voltage *U*_bias_ = *U*_DC_ + *U*_AC_ sin(ν_el_·*t*) three frequency components of the electrostatic interaction are obtained from Equation 2; a DC signal, a signal *U*_ν_ varying with the same frequency as the electrical modulation frequency ν_el_, and a signal *U*_2ν_ varying with twice that frequency [14–15,53–54]. These signals *U*_ν_ and *U*_2ν_ are analyzed using lock-in techniques to obtain the electrostatic images EAFM_ν_ and EAFM_2ν_. The first is related to the contact potential difference *U*_CP_ between tip and sample and the second to the capacitance *C*″(*d*) of the tip–sample system. To measure contact potential images the Kelvin technique is used: the signal EAFM_ν_ is nullified with an auxiliary feedback system by adjusting the tip voltage *U*_DC_, then the voltage applied to the tip is precisely the contact potential (*U*_DC_ = *U*_CP_). Frequency detection gives higher spatial resolution than force-detection electrostatic AFM, in addition, it allows for a correct determination of contact potential [42,55]. An external lock-in amplifier (Signal Recovery 7280 wide bandwidth DSP lock-in amplifier) was employed for the electrostatic AFM and KPM measurements using *U*_AC_ voltages as low as *U*_AC_ ≈ 500 mV at an electrical modulation frequency ν_el_ ≈ 7 kHz. Further details of how DAFM and Kelvin probe microscopy (KPM) [14] are implemented in our experiments is described elsewhere [42,50–52]. WSxM software was used for image processing [56]. Typically, a plane filter was applied to topography images; no filter is applied to the electrostatic images.

#### Interaction imaging

Multidimensional spectroscopy data is acquired as “interaction images” using the 3D-mode routine of the WSxM acquisition program [48,56–57]. As discussed in more detail elsewhere, for each tip–sample distance a parabola is obtained that shows a quadratic dependence of the frequency shift with bias, as expected from Equation 2. Fitting the experimental data to a parabola, for each horizontal (voltage) scan line three parameters are determined: the position of the minimum of each parabola, corresponding to the contact potential *U*_CP_, the vertical “offset” of the parabola (measured interaction at the minimum of the parabola) corresponding to the van der Waals interaction, and finally the curvature of each parabola, which is determined by the capacity, and thus by the strength of electrostatic interaction. For each interaction image three curves are obtained: a (true) van der Waals interaction Δν_vdW_(*d*) curve, the tip–sample capacitance *C*″(*d*) curve and a contact potential versus distance curve *U*_CP_(*d*). As discussed in more detail in [48], the algorithm to process the interaction images determines not only the tip radius from the term *C*″(*d*) = πε_0_*R*/(*d + h*/ε)^2^ and the product *A*·*R* from the Van der Waals interaction curve Δν_vdW_(*d*) = *A*·*R*/(3*d*^3^). In addition, also the poles of electrostatic and van der Waals interaction are determined, i.e., fits to algebraic functions 1/(*d* + *d*_poleEstat_)^−2^ and 1/(*d* + *d*_polevdW_)^−3^ are performed (see discussion in [48] for more detail). To obtain the Hamaker constant the experimental data is essentially processed as follows: First, a radius *R* and the pole of the electrostatic interaction *h*/ε are determined from the capacity curve *C*″(*d*) by fitting to the term πε_0_*R*/(*d* + *h*/ε)^2^ (second term in Equation 3). Then the Hamaker constant is determined from a fit of the van der Waals versus distance curve, Δν_vdW_(*d*), to the first term in Equation 3: *A*·*R*/(3*d*^3^), see Figure 3. To be more specific: essentially, our processing algorithm calculates the product *A*·*R* from the fit to Δν_vdW_(*d*). Then, using the tip radius *R* obtained from the capacity curve C″(*d*) the Hamaker constant *A* is determined uniquely from the product *A*·*R*. Note that this process is robust with regard to the tip size, since the van der Waals interaction is “normalized” to the tip radius by determining tip radius from the electrostatic interaction. In this sense, as discussed in the introduction (Derjaguin approximation, Equation 1) our technique allows us to separate tip geometry (*R*, determined through the electrostatic interaction) from “tip chemistry” determined through the van der Waals interaction. The methodology of “interaction imaging” is described in detail in [48]. A more detailed description of the methodology for the determination of the Hamaker constant will be presented elsewhere.

#### Sample preparation

For the experiments platinum-coated (on tip-side) silicon cantilevers (Olympus OMCL-AC240TM), silicon nitride tip-sharpened (Olympus OMCL-HA100) and all-in-one platinum-coated (on tip-side) silicon probes (Budget Sensors AIOAl-TL) were utilized. All the experiments have been performed at room temperature under ambient conditions. To clean the cantilevers the RCA process has been used [32]. In our experiments the two steps of the process are implemented as follows: The first step, Standard Clean-1 is performed with a solution composed of 5:1:1 parts by volume of Milli-Q water, NH_4_OH (ammonium hydroxide, 29%) and H_2_O_2_ (hydrogen peroxide, 30%). When utilized as sample, the chips with the cantilevers are sonicated for about 1 min in this solution, which removes organic residues. When used as cantilevers, the cantilever chips are immersed in the corresponding solution. Before the next step the samples are rinsed with Milli-Q water. The second step (Standard Clean-2) is performed with a solution composed of 5:1:1 parts by volume of Milli-Q water, H_2_O_2_ (hydrogen peroxide, 30%), and HCl (hydrochloric acid, 37%). Again, the cantilevers are sonicated (or immersed) for about 1 min in this solution and then rinsed with plenty of Milli-Q water. This second step removes metallic (ionic) contaminants that may have been deposited in the Standard Clean-1 cleaning step. In addition, for silicon surfaces this second step forms a thin passivating layer. Finally, the surface of the samples is dried by blowing with N_2_ for about 1 min.
